# Sunflower Meal Valorization through Enzyme-Aided Fractionation and the Production of Emerging Prebiotics

**DOI:** 10.3390/foods13162506

**Published:** 2024-08-10

**Authors:** Milica Simović, Katarina Banjanac, Milica Veljković, Valentina Nikolić, Paula López-Revenga, Antonia Montilla, Francisco Javier Moreno, Dejan Bezbradica

**Affiliations:** 1 Department of Biochemical Engineering and Biotechnology, Faculty of Technology and Metallurgy, University of Belgrade, Karnegieva 4, 11000 Belgrade, Serbia; dbez@tmf.bg.ac.rs; 2 Innovation Center of Faculty of Technology and Metallurgy, Karnegieva 4, 11000 Belgrade, Serbia; kbanjanac@tmf.bg.ac.rs (K.B.); mveljkovic@tmf.bg.ac.rs (M.V.); 3 Department of Food Technology and Biochemistry, Maize Research Institute, Slobodana Bajića 1, 11000 Belgrade, Serbia; valentinas@mrizp.rs; 4 Department of Bioactivity and Food Analysis, Food Science Research Institute CIAL (CSIC-UAM), Nicolás Cabrera 9, 28049 Madrid, Spain; p.lopez.revenga@csic.es (P.L.-R.); a.montilla@csic.es (A.M.); javier.moreno@csic.es (F.J.M.)

**Keywords:** sunflower meal, byproduct valorization, enzyme-aided fractionation, xylo-oligosaccharides, emerging prebiotics

## Abstract

Recently, there has been a burgeoning interest in harnessing the potential of biomass and industry byproducts for the development of novel products and materials. In particular, this study explored the efficient valorization of sunflower meal (SFM), an underutilized byproduct of the oil extraction industry, usually discarded or used as low-value animal feed through enzyme-aided fractionation, specifically targeting the extraction and conversion of its abundant carbohydrate component, xylan, into emerging prebiotic compounds—xylo-oligosaccharides (XOSs)—which are recognized as promotors of a healthy gut microbiome and overall human wellbeing. An enzymatic treatment using Alcalase^®^ 2.4 L was implemented for facilitating the recovery of a highly pure hemicellulosic fraction (92.2% carbohydrates) rich in *β*-(1→4)-linked xylose residues with arabinose and glucuronic acid substitutions (DP-xylan). A further enzymatic treatment of this substrate, using ROHALASE^®^ SEP-VISCO under optimized conditions (70 °C, pH 6, 0.005% *v*/*v* enzyme concentration), produced 52.3% of XOSs with a polymerization degree (DP) less than 20 after two hours. Further analyses demonstrated that the majority of the obtained product had a DP less than 6, predominantly consisting of di- and trisaccharides (XOS2 and XOS3) without the significant generation of xylose. These findings highlight the significant potential of SFM for the generation of valuable prebiotic compounds in a sustainable manner.

## 1. Introduction

Sunflower (*Helianthus annuus* L.) stands as one of the world’s most widespread cultivated oil crops, alongside soybean and rapeseed [1,2]. Its good nutritional properties make it widely utilized in the food sector [1]; however, its greatest economic importance comes from the high oil content in the seeds [3] and its potential for the production of a high-quality oil that represents one of the most preferable and widely used vegetable oils in the world [1,4]. Despite ongoing efforts to enhance the oil extraction process, a significant portion of solid residues (hulls, meals, and cakes) continues to be generated worldwide [5]. These oilseed residues are usually discarded as waste or utilized as a cheap component of animal feed for various livestock species, with a negligible application in the human diet [6]. Sunflower meal (SFM), the primary byproduct of sunflower oil extraction, holds great potential as a source of proteins, dietary fibers, phenolic compounds, and minerals yet remains underutilized [7]. Its profitable industrial application is currently limited due to its comparatively low protein content [8], high levels of undigestible carbohydrates [9], and finally the abundance of antinutritional factors [10], especially phenolics, which may form complexes with proteins, thus leading to reduced functionality and digestibility of meals [5,11]. Accordingly, there has been growing interest in their profitable reutilization aligning with the principles of circular economy and sustainable development. The valorization of SFM could result in the preparation of multiple products with added value for the food and pharmaceutical industries [11]. The most commonly utilized methods for the valorization of SFM are based on the extraction of protein fraction [8], since there is a constant increase in demand for relatively cheap and adequate protein sources [12]. This is not surprising, bearing in mind the nutritional and technological advantages of SFM proteins [13,14]. Nevertheless, in view of fully achieving biorefinery goals, the polyphenolic and carbohydrate fractions should also be considered for valorization. Phenolic compounds are frequently eliminated within the first steps of the protein isolation process, but recent data show that researchers are shifting towards their utilization upon gaining new knowledge about the utility of these fractions [10,15]. However, the systematic valorization of the carbohydrate fraction, which makes up to 52% of dry matter (DM), depending on the oil extraction process [16], is still insufficiently studied. Conventional extraction methods to valorize carbohydrate fractions in different byproducts and waste materials, based on multi-step extractions using acids and alkalis, come with many limitations, such as a loss in fiber functionality, laborious operation with low extraction efficacy, and, more importantly, environmental unacceptability, since the rigid lignocellulose structure requires severe pretreatments to facilitate the efficient extraction of the mentioned fractions [17]. Pectic compounds are usually extracted using mineral or organic acids [18,19], while traditional hemicellulose extraction methods are mostly based on the alkaline extraction (sodium or potassium hydroxides) of xylan with or without a delignification step [20,21,22]. Delignification is usually provided with sodium chlorite, hydrogen peroxide, or peroxyacetic acid, with the aim of obtaining purer xylan fractions, since alkaline treatment also partially solubilizes present lignin [23]. These methods have caused unavoidable hemicellulose degradation, and therefore, alternative means of isolation have been purposed, such as organic solvent treatments using different organic solvents (e.g., dimethyl sulfoxide, mixtures of dimethyl sulfoxide with other compounds and dioxane), but these methods have provided significantly lower yields of the wanted compounds, probably being restricted to the water-soluble fraction of xylan [24]. Additionally, the application of complementary emerging technologies such as microwaves, ultrasound, or enzymatic assistance is currently growing [25,26,27]. In the field of sunflower carbohydrate valorization, only sunflower heads were previously utilized for pectin extraction [28,29,30,31], while SFM was not subjected to either pectin or xylan extraction to date.

Xylan is commonly studied for textile, food, and biomedical applications; however, it has recently been widely utilized for the production of non-digestible oligomers, known as xylo-oligosaccharides (XOSs), a promising functional ingredient with diverse applications in the food and pharmaceutical industries. These xylose-based oligosaccharides are naturally occurring in vegetables, fruits, and honey [32] but in insufficient quantities. XOSs are viewed as promising emerging prebiotics that have a crucial role in maintaining gut health, and they are simultaneously well suited for incorporation into a wide variety of food and feed products. This is due to their exceptional application characteristics, including stability at high temperatures (up to 100 °C) and across a broad pH range (2.5–8), as well as their good sweetening power with minimal caloric impact [33]. XOSs may be produced using commercially available xylan; however, in order to enable their economically viable production nowadays, a major focus has been placed on the utilization of lignocellulosic biomass, which represents a cheap source of xylan [34]. To date, a large number of lignocellulosic materials (wheat and rice straw, wheat and barley brans, sorghum and grape stalks, sugarcane bagasse, corncob, beechwood, and birchwood) have been studied as xylan sources for XOS production with varying efficiencies, mostly depending on the type of xylan present and the conversion methods employed [35]. Therefore, different methods of XOS production and the great diversity in potential xylan substrates lead to a wide spectrum of different XOS structures with varied substituents of the xylose backbone and degrees of polymerization, which consequently have a great impact on its prebiotic and other functional properties.

The main aim of this work was to propose a promising pathway for SFM valorization, primarily through enzyme-aided extraction of xylan, which would be further used in the production of valuable xylo-oligosaccharides (XOSs) as emerging prebiotics. Thus, the enzymatic hydrolysis of the obtained xylans will be achieved using the highly promising enzymatic preparation Rohalase^®^ SEP-Visco, a thermostable bacterial xylanase from *Trichoderma reesei* that has not been previously applied for similar purposes. In this way, it is expected to provide value-added prebiotic-rich fraction of SFM.

## 2. Materials and Methods

### 2.1. Materials

The partially dehulled sunflower meal (SFM) utilized in this study was a kind donation from Victoriaoil LTD (Šid, Serbia). Analytical-grade chemicals, including ethanol, acetone, hydrochloric acid, sodium chlorite, sodium hydroxide, and ammonium acetate were obtained from Centrohem (Stara Pazova, Serbia). HPLC-grade solvents (acetonitrile and water), tri-fluoroacetic acid (TFA), hydroxylamine chloride, pyridine, hexamethyldisilazane (HMDS), pullulan standard set, phenyl-*β*-D-glucoside, and 1-phenyl-3-methyl-5-pyrazolone (PMP) were purchased from Sigma-Aldrich (Schnelldorf, Germany). Xylo-oligosaccharides (XOSs), standards (xylobiose, xylotriose, xylotetraose, xylopentaose, xylohexaose) were purchased from Megazyme LTD, Wicklow, Ireland. The enzymes used in the study were Alcalase^®^ 2.4 L, Novozymes (Bagsværd, Denmark), and Rohalase^®^ SEP-Visco which kind donation from AB Enzymes GmbH (Darmstadt, Germany).

### 2.2. Compositional Analysis of Sunflower Meal

The compositional analysis of sunflower meal was performed following the official AOAC methods for determining total dry matter, protein, lipids, and ash (950.01, 920.87, 920.39, and 923.03, respectively). Fiber content, namely neutral detergent fiber (NDF), acid detergent fiber (ADF), and acid detergent lignin (ADL), was determined using the Van Soest detergent method [36]. After filtering and drying, the NDF, ADF, and ADL were calculated as a percentage of the original sample. The content of hemicellulose was obtained as the difference between NDF and ADF content, while the cellulose content was calculated as the difference between ADF and lignin content. The content of total sugars, reducing sugars, and sucrose was determined by the Luff–Schoorl method [37].

### 2.3. Sunflower Meal Fractionation

#### 2.3.1. Method of Conventional Sunflower Meal Fractionation

**Figure 1:**
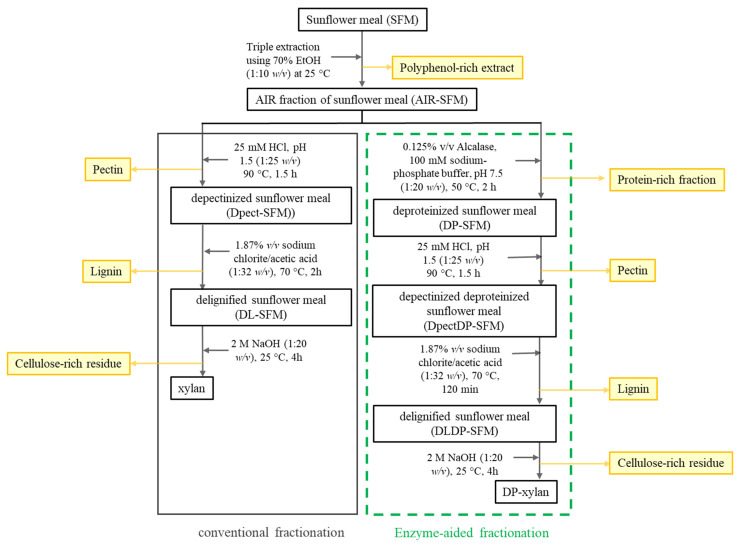
Schematic representation of applied SFM fractionation methods.

The schematic representation of the fractionation process applied to SFM is shown in Figure 1. The preparation of the alcohol-insoluble residue from sunflower meal (AIR-SFM) involved triple extraction with a 70% *v*/*v* aqueous ethanol solution at a 1:10 *w*/*v* ratio. The solid residue (AIR-SFM) was recovered via vacuum filtration and washed with 96% ethanol and acetone. The combined ethanol extracts were concentrated using a rotary vacuum evaporator, yielding a polyphenol-rich fraction. The next step in the fractionation process involved treatment with hot, diluted hydrochloric acid (HCl, pH adjusted to 1.5) at a 1:25 *w*/*v* ratio, heated to 90 °C for 1.5 h. The resulting extract was separated by vacuum filtration, and the solid residue was rinsed with hot water. Pectin was then precipitated from this mixture by cold ethanol precipitation overnight, using 4 volumes of 96% ethanol and recovered by centrifugation at 4427× *g* for 10 min. The solid residue (Dpect-SFM) was washed with 96% ethanol and acetone, then dried. The subsequent fractionation step was delignification, carried out using a 1.87% *w*/*v* solution of sodium chlorite with 1.87% *v*/*v* acetic acid at a 1:32 *w*/*v* substrate-to-solvent ratio at 70 °C for 2 h. The delignified solid residue (DL-SFM) was recovered by vacuum filtration, rinsed with water until neutral pH was reached, and then washed with 96% ethanol and acetone, followed by drying. The solubilized lignin was lyophilized. The final step in the xylan extraction process involved alkaline extraction with a 2 M sodium hydroxide solution at a 1:20 *w*/*v* ratio, with continuous mixing at room temperature for 4 h. The resulting extract was separated by vacuum filtration and adjusted to pH 6 using concentrated acetic acid, and then xylan was recovered by cold ethanol precipitation overnight with 4 volumes of 96% ethanol. The precipitate was collected by centrifugation at 4427× *g* and lyophilized to obtain xylan. The remaining solid residue, which is rich in cellulose, was rinsed with distilled water until neutral pH was achieved, then washed with 96% ethanol and acetone and dried. The obtained precipitates were recovered by centrifugation.

(1) $$Xylan\;yield\;(\%)=\frac{m\;(obtained\;xylan,\;g\;DM)}{m\;(SFM,\;g\;DM)}\times 100\%$$

(2) $$Xylan\;recovery\;yield\;(\%)=\frac{m\;(obtained\;xylan,\;g\;DM)}{m\;(hemicellulose,\;g\;DM)}\times 100\%$$

#### 2.3.2. Enzyme-Aided Method of Sunflower Meal Fractionation

Alcalase^®^ 2.4 L treatment of AIR-SFM was performed in a shaken Erlenmeyer flask (reaction volume of 500 mL) with an orbital shaker (120 rpm) at 50 °C. AIR-SFM was suspended in a 100 mM sodium-phosphate buffer (pH 7.5) at a ratio of 1:20 *w*/*v*, and the enzyme concentration was 2.5% (*v*/*w*), as calculated on AIR-SFM. In optimization experiments, one parameter was varied at a time in the following ranges: temperature: 40–60 °C; substrate-to-buffer ratio: 1:10–1:30 *w*/*v*; and enzyme concentration: 0.625–2.5% (*v*/*w*), as calculated on substrate. The concentration of proteins in supernatant was monitored for 2 h. At a predefined time, the aliquots of the reaction mixture were taken and incubated at 100 °C for 5 min to stop the reaction, centrifuged, and then analyzed by the determination of the protein concentration (mg eq BSA per 1 g of AIR-SFM) using the Lowry method [38].

A schematic representation of the enzyme-aided SFM fractionation process is shown in Figure 1. For the fractionation under the selected optimal conditions, AIR-SFM was suspended in 100 mM sodium phosphate buffer (pH 7.5) at a ratio of 1:20 *w*/*v*. The reaction was started with the addition of 2.5% of Alcalase^®^ 2.4 L, and the reaction was performed with constant mixing at 50 °C for 2 h. The solid residue from the reaction mixture (DP-SFM) was recovered by vacuum filtration and immediately rinsed with distilled water and afterwards washed using 96% ethanol and acetone to rinse the enzyme and stop the reaction, respectively. The protein-rich filtrate fraction was boiled at 90 °C to stop the reaction and afterward concentrated using the rotary evaporator and finally lyophilized to obtain the protein-rich fraction.

### 2.4. Obtained Xylan Analysis

#### 2.4.1. Fourier-Transform Infrared Spectroscopy (FTIR) Analysis

An FTIR analysis of extracted xylans was performed. KBr discs were prepared by mixing the xylan samples with KBr (1:100) and pressed. FTIR spectra were performed in a Bruker IFS66v equipment (Bruker, Bremen, Germany). Data were collected in absorbance mode using a frequency range of 4000–400 cm^−1^ and a resolution of 4 cm^−1^ (mid infrared region) with 250 co-added scans [26].

#### 2.4.2. Determination of Monomeric Composition

For the determination of the monomeric composition of SFM and the obtained xylans, a previously developed method was utilized [26]. Specifically, samples (30 mg) were hydrolyzed using 1.5 mL of 2 M TFA at 110 °C under inert conditions for 4 h. Upon hydrolysis, 300 μL of the hydrolyzed samples were evaporated. Afterwards, 400 μL of phenyl-*β*-D-glucoside internal standard (0.5 mg/mL) was added to each sample, and mixtures were evaporated again. The samples were thereafter derivatized and analyzed by GC-FID [26]. The analysis was carried out using an Agilent Technologies 7820A Gas Chromatograph (Agilent Technologies, Wilmington, DE, USA) equipped with a VF-5HT capillary column (5% phenylmethylpolysiloxane, 30 m × 0.250 mm × 0.10 μm; Agilent J&W, Folson, CA, USA). The injector temperature was 280 °C and the detector temperature was 385 °C. Nitrogen was used as the carrier gas with a flow rate of 1 mL/min. The temperature program was an initial temperature 120 °C and ramp of 3 °C/min up to 280 °C. Data acquisition and processing were carried out using Agilent ChemStation software Rev. B.04.03 (Wilmington, DE, USA). GC quantification was performed using *β*-phenyl-glucoside (0.5 mg/mL) as an internal standard and mixtures of the sugars of interest in a range of concentrations between 0.02 and 2 mg/mL.

#### 2.4.3. Determination of the Molecular Mass (Mw) Distribution by High-Performance Size-Exclusion Chromatography with a Evaporative Light Scattering Detector (HPSEC-ELSD)

The molecular weight (Mw) distribution was estimated according to a previously published procedure [29]. Samples (20 mg/mL) were prepared by dissolving them in distilled water for 30 min at an elevated temperature (50 °C), than mixed with mobile phase (0.04 M ammonium acetate) at a ratio of 1:9, filtered and separated by High-Performance Size Exclusion Chromatography with an Evaporative Light Scattering Detector (HPSEC-ELSD) (Agilent Technologies, Boeblingen, Germany) using the TSK-Gel guard column (6.0 mm × 400 mm) and two TSK-Gel columns connected in series, G5000 PWXL (7.8 mm × 300 nm, 10 μm), and G2500 PWXL (7.8 mm × 300 nm, 6 μm) (Tosoh Bioscience, Stuttgart, Germany). A pullulan standard set (Mw 0.342–788 kDa, 2–0.2 mg/mL) was utilized as the standard for the determination of molecular weight distribution.

### 2.5. Enzymatic Production of Xylo-Oligosaccharides (XOS)

The obtained xylans were finally utilized to synthesize XOSs in a shaken Erlenmeyer flask (reaction volume of 20 mL). The reaction mixtures were prepared by dissolving the obtained xylans in 100 mM sodium phosphate buffer (pH 6) to reach concentrations of 1% and 5% (*w*/*v*). The reaction was catalyzed by means of Rohalase^®^ SEP-Visco in a concentration range of 0.005–0.05% (*v*/*v*) at 50 °C with constant orbital shaking (120 rpm). Reactions were monitored for 2 h. At a predefined time, aliquots of the reaction mixture were taken and incubated at 100 °C for 5 min to stop the reaction. Parts of the samples were derivatized, filtered, and analyzed by high-performance liquid chromatography (HPLC), and selected samples were analyzed by means of HPSEC-ELSD and Matrix-Assisted Laser Desorption/Ionization Time-Of-Flight Mass Spectrometry (MALDI-TOF MS).

### 2.6. XOS Analysis

#### 2.6.1. Determination of Total Reducing Sugars

The total reducing sugars in the reaction mixture were determined using the method reported by Miller (1959) using the dinitrosalicylic acid reagent. The concentration of reducing sugars was calculated from the standard curve of xylose (1–10 mM) [39].

#### 2.6.2. High-Performance Liquid Chromatography (HPLC) Analysis of Reaction Mixture

Prior to HPLC analysis, samples were derivatized using the PMP in accordance with the procedure published by Wang et al. [40]. After derivatization, samples were filtered and analyzed using the Dionex Ultimate 3000 HPLC system (Thermo Fisher Scientific, Waltham, MA, USA) equipped with a reverse phase column (ZORBAX Eclipse Plus C18, 4.6 × 150 mm, 5 µm) at 30 °C using the mobile phase of 100 mM ammonium acetate buffer (pH 5.5):acetonitrile (80:20) with a constant flow rate of 0.5 mL/min [41]. The detection of products and standards was carried out using a UV detector at 245 nm. Data collection and processing were performed using Chromeleon 7.2. software. The concentration of the obtained compounds was calculated using the PMP-derivatized standard samples of XOSs (XOS2-XOS6).

#### 2.6.3. Matrix-Assisted Laser Desorption/Ionization Time-Of-Flight Mass Spectrometry Analysis (MALDI-TOF-MS)

The samples were diluted ten times in water and mixed at a 20:5 ratio (matrix:sample). The utilized matrix was 2,5-dihydroxybenzoic acid (DHB), with a concentration of 10 mg/mL in 90% aquatic methanol solution. All mixtures were treated with a strong cation exchange resin to eliminate present salts and avoid possible inhibition of the compounds of interest ionization. Analyses were carried out in positive ion detection mode in a range from 50 to 5000 Da, with the application of an ion deflection up to 400 to prevent mass matrix signals (which are the most intense) from saturating the detector. MALDI-TOF-MS analyses were performed on a Voyager DE-PRO mass spectrometer (Applied Biosystems, Foster City, CA, USA) at the Interdepartmental Investigation Service (SIdI-UAM) of Madrid.

#### 2.6.4. Statistical Analysis

All experiments were performed at least in duplicate, and the reported results were expressed as mean ± standard deviation. To determine the significant differences between the obtained results, the one-way analysis of variance (ANOVA) and Tukey’s post hoc test were applied using OriginPro 8.5. Differences at *p* < 0.05 were considered significant.

## 3. Results and Discussion

### 3.1. Chemical Characterization of Sunflower Meal

In the preliminary experiment, we thoroughly analyzed the compositional characteristics of partially dehulled sunflower meal (SFM), which served as the substrate for our study (Table 1 and Table 2). The dry matter (DM) of the SFM was found to be 91.5%, while the SFM was composed of 42.8% proteins, 47.0% carbohydrates, 2.8% fats, and 7.4% ash (all calculated on dry matter).

These results are in accordance with the ranges presented in previously published data on SFM, since the data on chemical composition vary considerably depending on the sunflower seed processing methods applied [42]. Specifically, dry matter (DM) values for different SFM ranged from 88.0 to 93.8%. Protein content was in the wide range of 26.4 to 40.3% per total weight, while the total mineral content was found to be rather constant (5.5 to 7.8%) in all examined samples [42]. On the other hand, the data concerning the detailed carbohydrate composition of SFM are quite scarce since most studies focus on the protein fraction of the meal [43]. Specifically, the majority of data about SFM carbohydrates are expressed through crude fiber (CF) content (11.5 to 29.7% [44]) or dietary fiber (DF) content (35.8–51.0% DM [43]). Carbohydrate analysis of dehulled SMF in our study revealed that they comprise simple sugars (5.5% DM), where sucrose content was predominant, with smaller amounts of monosaccharides (glucose and fructose) and oligosaccharides (most probably raffinose and stachyose [43]) and, on the other hand, a high amount of polysaccharides.

Among the mentioned polysaccharides, SFM contains 12.7% DM hemicellulose and 13.5% DM cellulose. The remaining portion (up to 8.5% DM) can likely be attributed to pectin, bearing in mind that the obtained results on SFM monomeric composition showed that galacturonic acid is present in moderate quantities at 14.35% of total monosaccharides (Table 2). Similarly, regarding the monomeric composition of SFM, it can be seen that xylose (21.8%), together with arabinose (21.1%) and glucose (22.3%), represents the most frequent monomeric building block for the polysaccharides present and therefore shows that SFM represents a highly potent substrate for xylan extraction.

### 3.2. Sunflower Meal Fractionation and Xylan Extraction

In the preliminary experiment, the extraction of xylan was performed using the most frequently utilized simple method of alkaline treatment for substrates rich in xylan, based on previously published literature data on xylan extraction [22]. However, this method proved to be unsuccessful since a product with low purity of isolated xylan was obtained (with 61.5% of DM being proteins), probably due to the complex composition of SFM that includes proteins and other carbohydrates. Specifically, this treatment causes a disruption of the complex structure by cellulose swelling and the hydrolysis of uronic and acetic acid esters linkages, as well as the dissolving of hemicellulose and lignin [44]. Additionally, these alkaline conditions simultaneously favor protein extraction [45]. Therefore, a more complex multi-step SFM fractionation approach to obtaining different carbohydrate fractions and protein isolate utilized for more complex substrates was approached (Figure 1).

The conventional method of SFM multi-step fractionation (Figure 1) included the primary removal of extractables (polyphenolic compounds, simple sugars, and colorants) using a 70% ethanol solution (Figure 1). The resulting extract proved to be rich in polyphenols (predominantly chlorogenic and caffeic acid), which could be applied as antioxidant agents in different formulations and potentially exhibit prebiotic activity on gut and skin microbiota [46].

The remaining solid fraction (AIR-SFM) had retained 80% of SFM DM, since simple sugars, polyphenols, and oils were separated by the extraction process. This fraction proved to be rich in proteins (51.0% of DM) and had approximately 38.0% of DM of carbohydrates. Since the presence of pectin in SFM was assumed, the step of pectin extraction was introduced according to the work of Cebin et al. [35] in order to make better utilization of all the present fractions. Also, bearing in mind that further steps (e.g., alkaline treatment) could impose structural and functional modifications on the native pectin structure [44], this step was performed at the beginning of the fractionation process. In this step, a pectin-containing fraction (14 g) was obtained (extraction yield of 15.34%), but it should be noted here that the obtained fraction might have a poor purity since a great amount of proteins (10 g) were extracted alongside pectin, which would introduce a need for further purification of the obtained fraction.

The remaining depectinized solid residue (DPect-SFM) was thereafter subjected to delignification treatment. This step was introduced since it was determined that SMF possesses around 6.8% of lignin calculated on DM that can be solubilized under alkaline conditions alongside xylan. For this treatment, sodium chlorite with the addition of acetic acid was chosen based on previously published data concerning its performance and selectivity [35]. Under optimized conditions, one-step delignification of DP-SFM was performed for 2 h and resulted in approximately 18 g of a lignin-containing fraction with a high quantity of proteins (11.7 g proteins). With this in mind, additional experiments were performed without the delignification step, yet a lack of this step highly affected the quality of the finally obtained xylan. Finally, the next step—alkaline treatment—was therefore performed on delignified solid residue (DL-SMF) using 2 M of sodium hydroxide (1:20) for 2 h at room temperature. This step yielded 6.5 g DM of xylan (yield of 7.1% and recovery yield of 56%) and around 34 g DM of cellulose-rich fraction. The obtained xylan had 52.5% carbohydrates (mainly composed of xylose at 53.7%) and 25.3% proteins, calculated on DM (Figure 1). Finally, it can be concluded that a high share of proteins ended up in different carbohydrate-rich fractions. Therefore, to prevent this protein loss, as well as the xylan yield and purity, a new method for fractionation was proposed. This method included the utilization of enzymes that will help with protein separation and enable the easier and more efficient fractionation of carbohydrates.

### 3.3. Optimization of Protein Extraction from AIR-SFM Using Alcalase^®^ 2.4 L

Generally, the extraction of proteins from lignocellulosic plant materials presents a great challenge. Consequently, an enzyme-aided extraction method is frequently proposed as an environmentally friendly technique that can be used to facilitate protein extraction from different plant sources. Enzymes simultaneously disrupt the cell wall and extract proteins by detaching the structural protein complexes from large polysaccharide matrices or/and degrading those to smaller molecular mass proteins and peptides [47]. To maximize the efficiency of protein extraction from lignocellulosic plant materials, the key process parameters, such as pH, temperature, reaction time, substrate, and enzyme concentration, should be determined.

**Figure 2:**
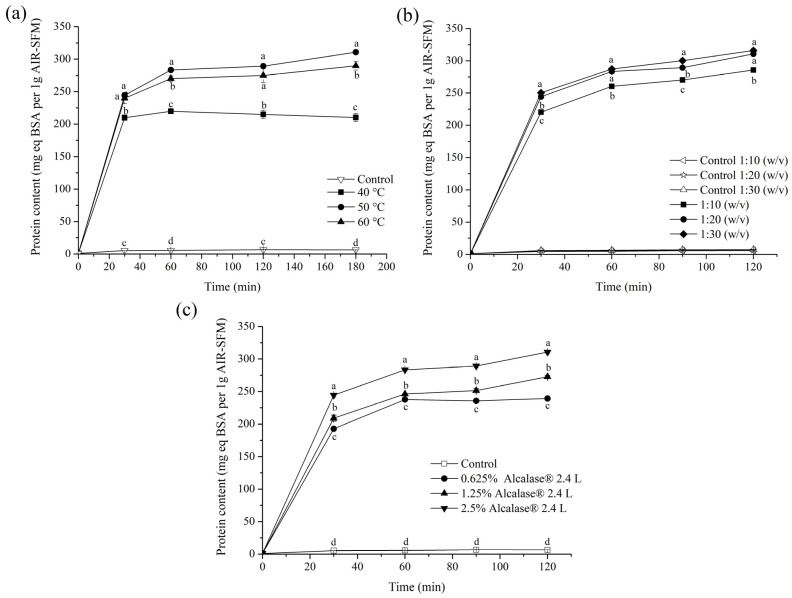
The influence of temperature (**a**), AIR-SFM-to-buffer ratio (**b**), and enzyme (Alcalase^®^ 2.4 L) concentration (**c**) on the enzyme-aided protein extraction process. Different characters on each graph at the same examination time indicate a statistically significant difference between (**a**) the control sample and temperatures, (**b**) the AIR-SFM-to-buffer ratios (it was statistically confirmed that each examined ratio is significantly different from its control), and (**c**) the control sample and different enzyme concentrations.

In this part of the study, commercial protease preparation (Alcalase^®^ 2.4 L) was employed to achieve the maximum protein extraction efficiency from AIR-SFM (containing 37.8 g proteins). First, the influence of temperature (40–60 °C) on the protein extractability was examined, since it could potentially provide positive effects on both the enzyme activity and the extractability of proteins. The other parameters were kept constant (AIR-SFM to 100 mM sodium phosphate buffer (pH 7.5) at a ratio of 1:20 with enzyme concentrations of 2.5% expressed on substrate). Even though the enzyme possesses a wide optimum range in alkaline medium (pH 6–10), a buffer pH of 7.5 was chosen since higher pH values may induce xylan and lignin extraction [48]. According to the presented results (Figure 2a), it can be seen that almost equal protein concentrations were obtained at 50 °C and 60 °C, while reactions at 40 °C showed lower extraction yields. Therefore, the next experiments will be performed at 50 °C. To promote the diffusion of proteins from AIR-SFM into the solution, an optimum amount of solvent has to be determined. By adding a large amount of solvent during the extraction process, the extraction efficiency will likely be increased, but excessive dilution of the product should be avoided because it increases downstream processing costs. Therefore, a study of the influence of the AIR-SFM-to buffer ratio on the extraction process was performed.

The obtained results indicate that when the ratio was increased from 1:10 *w*/*v* to 1:20 *w*/*v*, a slightly enhanced protein extraction efficiency was achieved (approximately 300 mg eq BSA per 1 g of AIR-SFM), while further ratio increments did not induce any changes in the obtained results (Figure 2b). Accordingly, an AIR-SFM to buffer ratio of 1:20 was selected for further experiments. Finally, the influence of enzyme concentration was examined. A typical protease dosage of 0.5 to 5% per g of lignocellulosic plant material could be found in several studies, where protease-aided protein was extracted from soybean, rapeseed, peanut, lupin, rice bran, and sunflower [49,50]. In the case of AIR-SFM treatment with Alcalase^®^ using different amounts of enzyme (0.625–2.5% on substrate), the highest protein concentration of 300 mg/g AIR-SFM was obtained using 2.5% Alcalase^®^ 2.4 L (Figure 2c).

### 3.4. Enzyme-Aided Fractionation of Sunflower Meal

Upon the determination of optimal conditions for protein removal from the AIR-SMF, the modified xylan extraction process was developed (Figure 1). After the removal of extractables and the enzyme-catalyzed deproteinization step, the 42.9 g DM of solid phase (DP-SFM) that contained 7.4 g proteins was obtained. It can be seen that the deproteinization step was highly successful since 30.5 g of proteins (corresponding to 80.54% of the total proteins of AIR-SFM) were removed from the protein-rich fraction. The subsequent step in the fractionation process involved depectinization under identical conditions to those used in conventional fractionation. This step of enzyme-aided fractionation enabled improved purity and yield of the pectin-rich fraction, since 12.8 g DM (extraction yield 14%) and only 4.6 g of proteins (35.6% of fraction) were obtained, in comparison with 14.3 g pectin-rich fraction with 10.1 g proteins (70% of fraction) using the conventional method. During the delignification step, around 2.5 g of DM was removed to obtain 27.5 g of DLDP-SFM, which was further subjected to xylan extraction. Finally, 5.6 g DM of DP-xylan was isolated. The yield of the enzyme-aided fractionation process was 6.1%, while the recovery yield was 47.8%. The obtained DP-xylan contained 92.2% of carbohydrates, mostly composed of xylose (63.4%), which was significantly higher than in the case of the conventionally obtained xylan. Additionally, DP-xylan contained approximately 7% of proteins (Table 2). These findings support the necessity of adding the deproteinization phase to the fractionation process, as the amount of protein impurities in the xylan product decreased from 25.5% to 7%, significantly improving the purity of the xylan that was obtained (Figure 1).

### 3.5. Characterization and Comparison of Obtained Xylans

In view of summarizing the advantages of the newly developed method for SFM fractionation and xylan extraction, a more thorough comparison of the obtained xylan fractions is presented in Table 3, displaying the most important parameters. Thus, slightly higher quantities and accordingly higher yields of the obtained xylan fractions were achieved with the conventional fractionation method. These results are expected and in accordance with previously published data. For example, Sporck et al. showed that the enzymatic approach gave 2.4 times lower yields than the alkaline method due to the higher number of extraction steps and the extraction selectivity [17].

However, higher yields using the conventional method are obtained at the expense of the purity of the samples, which can be seen when it comes to the carbohydrate and protein content. Specifically, conventionally obtained xylan has a carbohydrate content of 52.5% DM, corresponding to a total amount of 3.4 g of carbohydrates, while, on the other hand, DP-xylan, obtained according to the newly proposed method, had a significantly higher carbohydrate amount (92.2% DM), implying a greater purity. This corresponds to 5.1 g of carbohydrates, which is almost 1.5 times more than in the previous case. In terms of the achieved recovery yields, the results obtained in this study were better than in the case of alkaline-sulfite-pretreated sugarcane bagasse, where the recovered yields of 53% and 22% for conventional and enzymatic treatment were achieved, respectively [17]. Likewise, similar yields (4.5–8.5%) were obtained by Rowley et al. using delignified corn stover by means of different methods [24].

**Figure 3:**
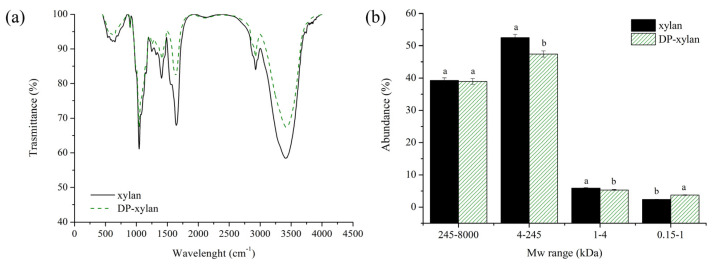
FTIR spectra (**a**) and distribution of molecular weights (Mw) (**b**) of the obtained xylans. Different characters on each MW range indicate a statistically significant difference between xylans.

The analyzed infrared spectra of the both obtained xylans show characteristic bands for xylan-rich compounds (Figure 3a). Specifically, bands occurred at 3414 cm^−1^, which can be assigned to the stretching vibrations of the O-H groups, and bands occurred at 2926 cm^−1^, which are generally assigned to the -CH_2_ antisymmetric stretching, while the band at 2850 cm^−1^ was a result of -CH_2_ symmetric stretching [51]. Bands occurring at 1644 cm^−1^ can be assigned to the absorbed water [52]. The difference between two xylan samples can be seen around 1510 cm^−1^ due to the aromatic skeletal vibration [53]. This band is characteristic for aromatics, which may originate from both proteins and lignin, and the fact that they are still visible in FTIR spectra can imply that proteins and lignin are not completely removed from the material. The bands occurring at 1450 cm^−1^ in some samples could be assigned to the presence of the methyl groups, while spectral peaks that are visible at 1044 cm^−1^ originated from C-O stretching in the C-O-C ether linkages. The peaks at 897 cm^−1^ can be attributed to the stretching vibration modes (both symmetric and antisymmetric) of C-O in the ether linkage and can prove the *β*-configuration of 1→4 glycosidic bonds between xylose units of the xylan chain [51]. Other bands at lower wavenumbers, such as 690 cm^−1^, are attributed to the out-of-plane C-H deformations [51]. The signals around 1249 cm^−1^ and 1736 cm^−1^ indicate the vibrational band of the single-bond C-O stretching band and C=O stretching related to the acetyl groups present in the xylan, respectively [54].

Furthermore, Figure 3b displays the estimated distribution of molecular weights (Mw) along with the corresponding relative abundances (%). Within the four domains, it is evident that the two obtained xylans—xylan and DP-xylan—have comparable Mw distributions. The two fragments with very broad Mw ranges (245–8000 kDa and 4–245 kDa) were the major ones, and they were followed in distance by two fragments with narrow ranges (between 1 and 4 kDa and 0.15–1 kDa), which corresponded to mono- and oligosaccharides. These results show that the introduction of the deproteinization step did not have a negative influence on the structure of extracted xylan.

### 3.6. Hydrolysis of Obtained Xylans toward Xylo-Oligosaccharides Production

After the extraction and characterization of SFM xylans, the final step in SFM valorization was to obtain emerging prebiotics, XOSs, by means of enzymatic conversion. For this purpose, bacterial xylanase Rohalase^®^ SEP-Visco was chosen as the optimal commercial preparation owing to the fact that it produces negligible amounts of xylose (preliminary study), thus ensuring the production of complex XOS mixtures without the need for extensive purification. This enzyme was applied for the first time for XOS production, and therefore, the first experiments enabled insight into the effects of the reaction factors needed for XOS production.

**Figure 4:**
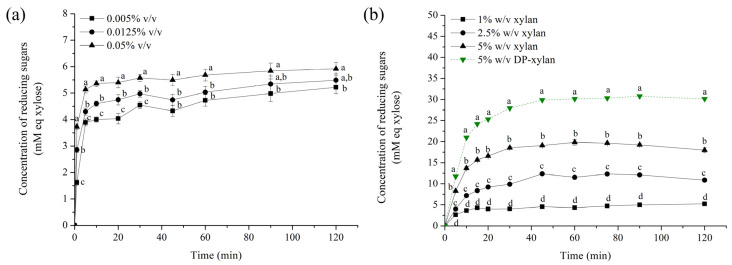
Time course of XOS production using Rohalase^®^ SEP-Visco: (**a**) Influence of enzyme concentration on hydrolysis of xylan; (**b**) influence of substrate concentration of xylan and DP-xylan. Different characters on each graph at the same examined time point to a statistically significant difference between (**a**) enzyme concentrations and (**b**) substrate concentrations.

It was concluded that optimum conditions for XOS production were a slightly acidic to neutral reaction medium, while the optimum temperature was found to be around 70 °C (Figure S1). After the determination of these process parameters, the influence of substrate and enzyme concentrations on XOS production was examined. As can be clearly seen from the time course graph (Figure 4a), the enzyme preparation proved to have significantly high activity and even small amounts of the enzyme (0.005% *v*/*v*) can be utilized for this purpose. Almost consistent maximum XOS concentrations were achieved in all experiments for rather short reaction times (after 2 h), regardless of the enzyme concentration. The only significant difference could be detected in terms of initial reaction velocities. Hence, the lowest examined enzyme concentration was adopted as optimal for the next set of experiments featuring higher concentrations of the substrate in order to examine the initial kinetics of the XOS synthesis reaction and accordingly to see differences in their structures throughout the whole reaction time course. As was expected, higher concentrations of XOSs were achieved, with an increment in the offered substrate concentration, reaching concentrations of reducing sugars of approximately 20 and 31 mM for conventionally and enzyme-aided extraction of xylan (DP-xylan), respectively, when a 5% (*w*/*v*) substrate solution was utilized. The obtained concentration did not exactly match the increment of the substrate (Figure 4b); however, this can be explained by the fact that higher xylan concentrations decreased XOS yields due to the enzyme inhibition caused by the higher reaction mixture viscosity and density, which was previously noticed by Bian et al. [55]. Nevertheless, it can be concluded that purer DP-xylan represents the substrate of choice for XOS production being able to yield significantly more XOSs. The results presented in this way (which overall produced reducing sugars) give us a comprehensive picture of the XOS reaction process; however, in order to better see what type of compounds were synthesized, as well as the degree of compound polymerization, additional characterization of the obtained reaction mixtures for DP-xylan was performed.

**Figure 5:**
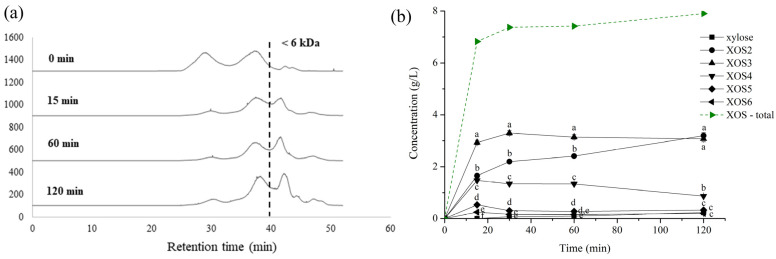
(**a**) HPSEC-ELSD chromatograms of XOS production from DP-xylan for reaction times 0, 10, 60, and 120 min. The vertical line represents 6 kDa (DP of approximately 50). (**b**) Time course of XOS production with achieved concentrations for compounds up to XOS6, analyzed using HPLC-UV. Different characters on the graph at the same examined time indicate a statistically significant difference between the concentrations of individual XOSs.

Specifically, HPSEC-ELSD was used to ascertain the ranges of the polymerization degree from the acquired XOSs. For this purpose, reaction mixtures for the reaction times 0, 15, 60, and 120 min were chosen for further analysis. It can be clearly seen that the hydrolysis of high-molarity DP-xylan (Figure 5a) occurs through the derivation of oligosaccharides (Mw < 6 kDa, corresponding to DP < 50). Specifically, the abundance of oligosaccharides lower than 6 kDa was around 10% in the initial reaction mixture (0 min), while the oligosaccharide abundance increased with the reaction time, reaching 43.0%, 48.3%, and 53.2% after 10, 60, and 120 min, respectively.

In order to quantify the obtained products, HPLC analysis was performed to analyze the concentration profile of oligosaccharides up to DP 6. From Figure 5b, it can be seen that the total XOSs rather quickly (15 min) reach an equilibrium concentration of 7.5 mg/mL, which represents an XOS yield of approximately 15% (calculated on total DP-xylan content). This concentration was higher than previously reported, 5.29 mg/mL, after 12 h using xylan-rich fraction isolated from sugarcane bagasse [54] and 5.05 mg/mL from xylan isolated from oil palm empty fruit bunch fibers after 12 h [55], but lower than 28.6% of xylan extracted from corncobs reached by Teng et al. [56] and 19.1% of XOSs from sugarcane bagasse by Valladares-Diestra et al. [57]. Significant discrepancies in the achieved yields are mainly a consequence of the xylan structure and composition, which depend on the type of employed extraction treatment, as well as the utilized enzyme. Besides the concentration, the composition of the obtained XOSs may vary greatly between different systems, as well as under different conditions within the same system. During the reaction of DP-xylan hydrolysis, the main reaction products and their fraction change while the XOS total concentration remains constant (Figure 5b). At the starting stages of the reaction, XOS3 seems to be the most abundant XOS derivative, while the XOS2 concentration increases during the whole examined reaction period, finally reaching the same concentration after 2 h. On the other hand, the concentration of XOS4 decreases over time, together with the less abundant XOS5 and XOS6. Once again, it should be emphasized that the concentration of xylose is quite low, owing to the fact that an enzymatic preparation with pronounced endo-xylanase activity was utilized.

**Figure 6:**
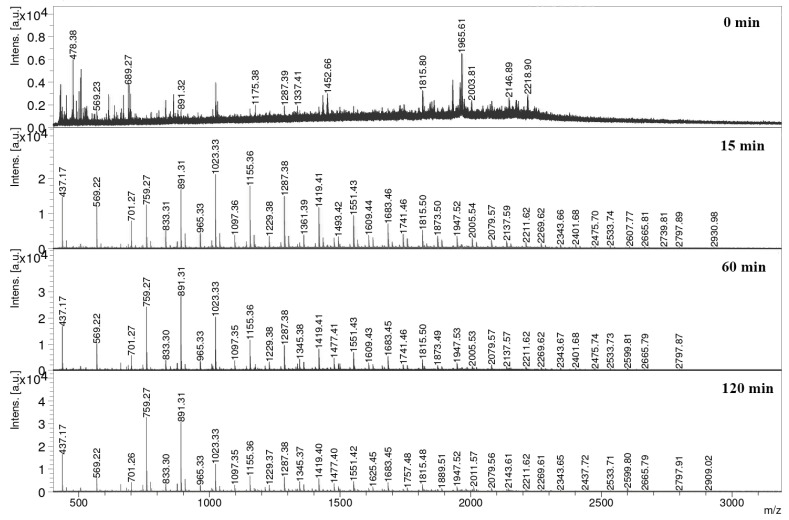
MALDI-TOF MS analysis of DP-xylan reaction mixture in various reaction times.

Even though oligosaccharides are often regarded as a group of diverse compounds with a degree of polymerization between 2 and 50 monomeric units or Mw values up to 5–7 kDa, according to different criteria [58], information about their composition is quite critical for the further establishment of structure–function relationships. According to the MC (Table 2), the main type of extracted hemicellulose was xylan, with the common feature of a backbone of *β*-(1→4)-linked xylose residues, with a common modification of xylans with numerous arabinose and some glucuronic acid residues on O-2 [59]. Therefore, the obtained structures were additionally characterized by means of MALDI-TOF-MS analysis, since both the chain length and the expected types of glycosidic linkage contained in the XOSs can be inferred. Specifically, the mass spectra confirmed the existence of pentose-based oligosaccharides DP3-DP22 as sodium adducts (*m*/*z* 437, 569, 701, 833, 965, 1097, 1229, 1230.0, 1362.1, 1494.2, 1626.3, 1758.4, 1890.6, 2022.7, 2154.8, 2286.9, 2419.0, 2551.1) and as potassium adducts (*m*/*z* 453.4, 585.6) with a lower abundance. These most likely correspond to xylose, according to the compositional data of DP-xylan (Table 2), although some of the xylose units may be substituted with arabinose, which cannot be substantiated by MALDI-TOF because xylose and arabinose have equal masses. None of these pentoses seem to be substituted with acetyl groups, which is in good agreement with the lack of a characteristic band for acetyl groups previously observed in FTIR spectra. It should be noted that oligomers below *m*/*z* 400 are not included in the spectrum due to hindrance of matrix peaks, and therefore, this analysis cannot confirm the presence of xylobiose and the small quantities of xylose that were previously detected by HPLC analysis. Thereafter, it can be seen from Figure 6 that the most important obtained compounds are acidic oligosaccharides, that are present in three series, (i) the most simple pentoses with one molecule of glucuronic acid with DP 5–17; (ii) the most abundant series are oligosaccharides (methylglucuronic acid (MeGlcA) substituted XOS), where DP4-DP21 (with *m*/*z* values of 628, 759, 891, 1023, 1155, 1287, 1419, 1551, 1683, 1815, 1947, 2079, 2212.8 2344.9, 2477.0, 2609.1, 2741.2, and 2873.3) are most likely present, and the potassium adducts with DP between 5 and17 (775.7, 907.8, 1039.9, 1172.0, 1304.1, 1436.2, 1568.3, 1700.5, 1832.6, 1964.7, 2096.8, 2228.9, and 2361.0) are also present; and (iii) XOSs with two groups of MeGlcA with a DP of 8–21 (1213.9, 1346.0, 1478.1, 1610.2, 1742.4, 1874.5, 2006.6, 2138.7, 2270.8, 2402.9, 2535.0, 2667.1, 2799.2, and 2931.3). Finally, in a small amount, a series of hexose oligomers (from DP 3 to DP 17) can be recognized within the spectrum (with *m*/*z* values of 527, 689, 851, 1031, 1175, 1333, 1499, 1661, 1823, 1824.6, 1986.7, 2148.8, 2311.0, 2473.1, 2635.3, and 2797.4), which might be a consequence of a small share of adjuvant polysaccharide fractions within the obtained DP-xylan.

## 4. Conclusions

Sunflower meal, an abundant and insufficiently exploited byproduct of the oil industry, has proven to be a good source of valuable compounds that could be valorized through the newly proposed efficient method based on enzyme-aided fractionation. The application of enzymes not only enabled an increase in the yield of purer xylan but also generated several other fractions (polyphenol-rich fraction, protein isolate, pectin, and lignin) that are ready to be used to add value to different food and cosmetic preparations. Finally, the high purity of the DP-xylan extract obtained was successfully transformed into an emerging group of prebiotic compounds, xylo-oligosaccharides (XOSs). Additionally, our enzyme-aided fractionation method will deliver other fractions enriched in other compounds, such as polyphenols, proteins, pectin, lignin, and cellulose, which can be utilized in the future as food additives or in the field of nutraceuticals and functional cosmetic ingredients.

## Figures and Tables

**Table 1:** Chemical composition of partially dehulled SFM. All data are presented as mean value ± standard deviation (analyses were performed in duplicate).

Component	Concentration
Dry matter (%)	91.5 ± 0.3
Simple sugars (% DM)	5.5 ± 0.3
Hemicellulose (% DM)	12.7 ± 0.9
Cellulose (% DM)	13.5 ± 0.1
Lignin (% DM)	6.8 ± 0.5
Proteins (% DM)	42.8 ± 0.1
Fats (% DM)	2.8 ± 0.0
Ash (% DM)	7.4 ± 0.1

**Table 2:** Monomeric composition of partially dehulled SFM polysaccharides (% total identified monosaccharides). DP-xylan: deproteinized xylan. All data are presented as mean value ± standard deviation. Different characters (a, b, and c) in the same row signify a statistically significant difference between substrates.

Component	Concentration		
	SFM	Xylan	DP-xylan
Xylose	21.8 ± 1.9 ^c^	53.7 ± 5.4 ^b^	63.4 ± 1.1 ^a^
Arabinose	21.1 ± 0.8 ^a^	11.2 ± 0.1 ^b^	9.7 ± 0.9 ^b^
Rhamnose	3.6 ± 0.2 ^a^	3.4 ± 0.3 ^a^	3.3 ± 0.1 ^a^
Fructose	2.4 ± 0.4 ^a^	1.1 ± 0.6 ^b^	1.7 ± 0.1 ^a,b^
Galactose	10.6 ± 0.4 ^a^	11.8 ± 7.3 ^a^	5.8 ± 0.6 ^a^
Mannose	3.9 ± 0.3 ^a^	2.0 ± 0.2 ^b^	0.5 ± 0.1 ^c^
Glucose	22.3 ± 2.9 ^a^	10.4 ± 1.1 ^b^	8.0 ± 0.2 ^b^
Galacturonic acid	14.4 ± 0.4 ^a^	6.5 ± 0.5 ^c^	7.7 ± 0.1 ^b^

**Table 3:** Characterization of obtained xylans. All data are presented as mean value ± standard deviation. Different characters in the same row signify a statistically significant difference between xylans.

Characteristic	Xylan	DP-xylan
Quantity (g)	6.7 ± 0.3 ^a^	5.8 ± 0.6 ^a^
Dry matter (%)	96.8 ± 1.1 ^a^	95.0 ± 2.2 ^a^
Yield (%)	7.1 ± 0.5 ^a^	6.1 ± 0.6 ^a^
Recovery yield (%)	56.0 ± 2.2 ^a^	47.8 ± 0.9 ^b^
Carbohydrate content (%)	52.5 ± 2.3 ^b^	92.2 ± 3.3 ^a^
Protein content (%)	25.3 ± 1.2 ^a^	7.1 ± 1.1 ^b^

## Data Availability

The original contributions presented in the study are included in the article/Supplementary Materials, further inquiries can be directed to the corresponding author.

## Supplementary Materials

The following supporting information can be downloaded at https://www.mdpi.com/article/10.3390/foods13162506/s1: Figure S1: (a) Influence of reaction temperature on XOS production, (b) Influence of reaction temperature on XOS production.
