# Supplementary material for: Sunflower Meal Valorization through Enzyme-Aided Fractionation and the Production of Emerging Prebiotics
Source: Foods. 2024 Aug 10;13(16):2506. doi: 10.3390/foods13162506 (PMC11353406; doi:10.3390/foods13162506)
Supplement: Supplementary file 1 [file foods-13-02506-s001.zip › foods-3113581-supplementary.pdf]

Supplementary material

for

Sunflower meal valorization through enzyme-aided fractionation and production of emerging prebiotics

Milica Simović[1*] , Katarina Banjanac[2], Milica Veljković[2], Valentina Nikolić[3], Paula López-Revenga[4], Antonia Montilla[4], Francisco Javier Moreno[4], Dejan Bezbradica[1]

[1] Department of Biochemical Engineering and Biotechnology, Faculty of Technology and Metallurgy, University of Belgrade, Karnegieva 4, 11000 Belgrade, Serbia; dbez@tmf.bg.ac.rs
[2] Innovation Center of Faculty of Technology and Metallurgy, Karnegieva 4, 11000 Belgrade, Serbia; kbanjanac@tmf.bg.ac.rs (K.B.); mveljkovic@tmf.bg.ac.rs (M.V.)
[3] Department of Food Technology and Biochemistry, Maize Research Institute, Slobodana Bajića 1, 11000 Belgrade, Serbia; valentinas@mrizp.rs
[4] Department of Bioactivity and Food Analysis, Food Science Research Institute CIAL (CSIC-UAM), Nicolás Cabrera 9, 28049 Madrid, Spain; p.lopez.revenga@csic.es (P.L.-R.); a.montilla@csic.es (A.M.); javier.moreno@csic.es (F.J.M.)

[*] Corresponding author: Milica Simović, Faculty of Technology and Metallurgy, University of Belgrade, Karnegijeva 4, 11000 Belgrade, Serbia. E-mail address: mcarevic@tmf.bg.ac.rs. Tel.: (+38111)3303727

Enzymatic conversion of xylane into xylo-oligosaccharides (XOS)

To determine optimum conditions for XOS production, the influence of temperature (40-80 °C) and pH value of reaction mixture (pH 3-8) on xylan conversion was examined. The reaction mixtures were prepared by dissolving the xylan in corresponding buffer to reach concentrations of 1% (w/v). The reaction was catalyzed by means of Rohalase® SEP-Visco in a concentration range of 0.01% (v/v) at corresponding temperature. All reactions were performed in an Erlenmeyer flask (the reaction mixture volume was 10 mL) on an orbital shaker (IKA® KS 4000i control, Werke GmbH and Co., Staufen, Germany) at 120 rpm. At predefined times, samples were taken, diluted ten times with distilled water, treated in a Thermomixer (Eppendorf, Hamburg, Germany) for 5 min at 100 °C (for enzyme deactivation), then filtered and analyzed by DNS method. All experiments were carried out in triplicate, and average values are presented throughout the study. All standard deviations were less than 10%.

Determination of total reducing sugars – DNS method

The total reducing sugars in the reaction mixture were determined using the method reported by Miller (1959) using the dinitrosalicylic acid reagent. The concentration of reducing sugars was calculated from the standard curve of xylose (1-10 mM) [1].

Figure S1. (a) Influence of reaction temperature on XOS production, (b) influence of reaction temperature on XOS production

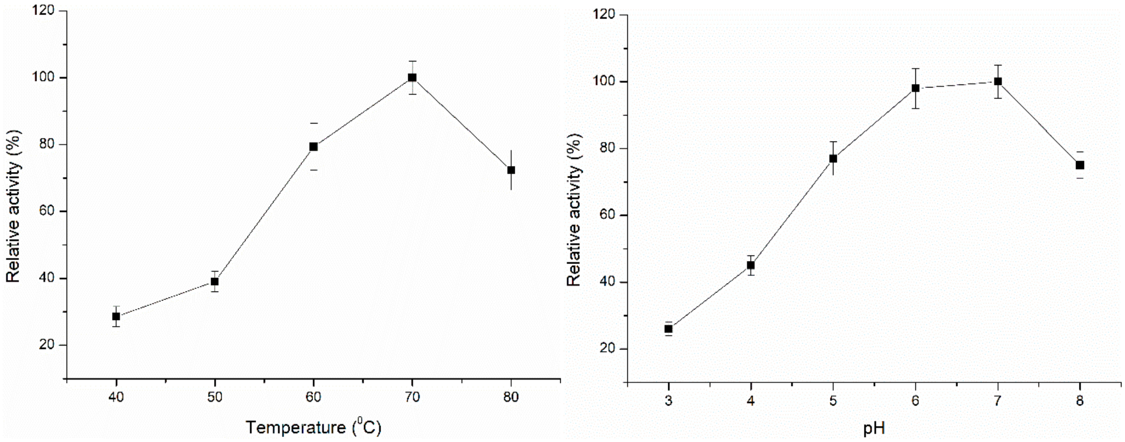

(a) **(b)**

References:

1. Petrov Ivanković, A.; Milivojević, A.; Ćorović, M.; Simović, M.; Banjanac, K.; Jansen, P.; Vukoičić, A.; van den Bogaard, E.; Bezbradica, D. In vitro evaluation of enzymatically derived blackcurrant extract as prebiotic cosmetic ingredient: extraction conditions optimization and effect on cutaneous microbiota representatives. Chemical and Biological Technologies in Agriculture 2023, 10, 125, doi:10.1186/s40538-023-00502-8.
